# How and when does artificial intelligence use enhance student creativity? The mediating role of knowledge-based dynamic capabilities

**DOI:** 10.3389/fpsyg.2026.1861541

**Published:** 2026-07-27

**Authors:** Huainan Wang, Feng Fu, Lei Li, Ming Liu

**Affiliations:** Business School, Chengdu University of Technology, Chengdu, China

**Keywords:** artificial intelligence self-efficacy, artificial intelligence trust, artificial intelligence use, knowledge-based dynamic capabilities, student creativity

## Abstract

As artificial intelligence (AI) becomes more deeply integrated into higher education, understanding its effects on student creativity has become increasingly important. However, the mechanisms through which AI use is associated with student creativity, as well as the boundary conditions of this relationship, remain insufficiently understood. Drawing on the knowledge-based dynamic capabilities (KBDC) framework, this study develops and tests a model that links AI use to student creativity while incorporating the moderating roles of AI self-efficacy and AI trust. Using survey data from 764 university students in China, we employ hierarchical regression analysis to examine the proposed model. The results show that AI use is positively associated with student creativity and that this relationship is mediated by students’ knowledge acquisition, absorptive, and combination capabilities. Furthermore, AI self-efficacy strengthens the positive effects of AI use on all three capability dimensions, whereas AI trust weakens the effects on knowledge acquisition and absorptive capabilities. In doing so, this study advances research on AI use and student creativity by unpacking one important capability-based mediating pathway and the boundary conditions under which this relationship is stronger or weaker. It also contributes to the KBDC literature by extending the application of KBDC theory from the organizational to the individual level and demonstrating how these capabilities operate in AI-enabled learning contexts. The findings offer practical implications for educators, universities, students, and policymakers seeking to foster creative and reflective AI-supported learning.

## 1 Introduction

Creativity refers to the capacity to generate ideas that are both novel and effective (Runco and Jaeger, 2012). It has been widely recognized as a core twenty-first-century skill, actively promoted in education systems globally (Marrone et al., 2022), and valued across a broad range of disciplines such as business, science, and the arts (Habib et al., 2024). Reflecting this growing emphasis, universities have increasingly embedded creativity development into their curricula through dedicated courses and training programs. Against this backdrop, our study conceptualizes creative students as those who can effectively recognize problems, integrate diverse information, and generate original and valuable ideas and solutions.

As higher education places increasing importance on student creativity, the rapid advancement and widespread adoption of artificial intelligence (AI) have become one of the most prominent developments shaping contemporary universities. Artificial intelligence can be broadly defined as the capacity of systems to interpret external data, learn from such data, and apply acquired knowledge to achieve specific goals and tasks (Haenlein and Kaplan, 2019). AI use has become an integral part of students’ everyday academic practices. A survey conducted in the United States reported that 89% of college students used ChatGPT to complete assignments, while 53% relied on it for essay writing (Yu, 2023). AI technologies are transforming educational practices by introducing new ways of information processing, problem solving, and decision making (Mazhar and Arseven, 2026).

Given the widespread diffusion of AI technologies and the central role of creativity in higher education, scholars have increasingly examined the relationship between AI use and student creativity, yet the findings have been mixed. On the one hand, a stream of research underscores the capacity of AI tools to support creative processes (Chan and Hu, 2023; Habib et al., 2024). By drawing on extensive knowledge bases to introduce diverse perspectives, AI tools can prompt students to question existing assumptions, stimulate curiosity, encourage thinking outside the box, and ultimately inspire more creative thinking (Dai et al., 2023). On the other hand, scholars have cautioned that inappropriate use of AI may undermine student creativity (Rasul et al., 2023). The availability of instant, AI-generated answers may foster technological dependence and reduce students’ opportunities for independent thinking and active exploration (Wang and Li, 2023). Furthermore, overreliance on AI may weaken students’ capacity for original thought and ultimately constrain creativity (Mazhar and Arseven, 2026). These conflicting voices regarding the effects of AI are further supported by studies that examine students’ own perceptions. For instance, in a survey of 221 university students in Croatia, 31% agreed that AI use enhances creativity, 32% disagreed, and 36% remained neutral (Stadnik et al., 2025).

The growing body of research on AI use and student creativity has yielded mixed findings, indicating that the relationship is unlikely to be simple or direct. Notably, AI can provide students with external knowledge inputs in the form of information, advice, explanations, answers to questions, and predictive insights, but these inputs do not by themselves constitute creativity (Glikson and Woolley, 2020). Creativity, however, is fundamentally a knowledge-intensive process (Bensalah and Mâţă, 2022; Teodoridis et al., 2019). AI-provided inputs are more likely to support creativity when students engage in a knowledge-based capability-building process that uses AI to acquire relevant information and insights, broaden knowledge diversity during brainstorming, and develop promising ideas into novel outputs (Baltà-Salvador et al., 2025; Huang et al., 2025).

In other words, a key to explaining the AI use–student creativity relationship lies not simply in whether students use AI, but in whether they can transform AI-provided inputs into creative outcomes through a knowledge-conversion process. Yet existing research has rarely explained this relationship from that perspective. We argue that the knowledge-based dynamic capabilities (KBDC) framework is particularly well suited to address this gap. This is because KBDC refers to the ability to acquire, generate, and combine knowledge to sense and respond to environmental changes (Zheng et al., 2011), and thus captures a knowledge-based capability-building process through which external knowledge inputs are transformed into innovative outcomes (Kaur, 2023; Zheng et al., 2011).

Therefore, we draw on the KBDC framework in this study to examine to what extent AI use enhances student creativity through the development of knowledge-related capabilities. Building on prior KBDC research, we focus on three core capability dimensions relevant to explaining creative outcomes—knowledge acquisition, knowledge absorptive, and knowledge combination capabilities (Denford, 2013; Kaur, 2023; Zheng et al., 2011). Furthermore, a resource-based perspective suggests that the value and effects of AI use depend on individuals’ ability and willingness to use AI effectively (Liang et al., 2010). Accordingly, at the individual level, we introduce students’ AI self-efficacy and AI trust as boundary conditions to examine how they moderate the relationship between AI use and KBDC.

This study makes three theoretical contributions. First, we advance research on AI use and student creativity by proposing and testing the mediating role of KBDC. Rather than assuming a direct association between AI use and student creativity, our findings suggest that this relationship operates through three dimensions of KBDC: knowledge acquisition, knowledge absorption, and knowledge combination. This suggests that AI may support student creativity not by substituting for independent thinking, but by strengthening students’ active engagement with knowledge. Second, our study identifies important boundary conditions by examining whether these relationships vary according to students’ AI self-efficacy and AI trust. This provides a more nuanced understanding of when AI use might be more or less beneficial for student creativity.

Third, this study contributes to the KBDC literature by extending its application from the organizational to the individual level and by situating it in an AI-enabled learning context. By examining how KBDC operates in students’ use of AI, the study highlights AI not simply as an information source, but as a tool whose creative value depends on how effectively students leverage it. These insights also have practical implications for students, educators, and higher education institutions, underscoring the importance of fostering active and reflective uses of AI rather than passive reliance on AI-generated outputs.

## 2 Literature review and hypothesis development

### 2.1 AI use and student creativity

Drawing on Runco and Jaeger (2012) definition of creativity, we define student creativity in the present study as students’ ability to generate ideas or solutions that are both novel and useful in learning contexts. In such contexts, creativity plays a central role by enabling students to navigate challenges and find solutions in any fields (Habib et al., 2024).

AI use refers to students’ use of AI technologies, such as ChatGPT, to support learning tasks and academic work. AI tools, powered by advanced natural language processing and extensive knowledge bases, have been shown to enhance learning processes (Dai et al., 2023) and innovation-related outcomes (Liu C. et al., 2026a). Notably, prior research suggests that the relationship between AI use and student creativity is not uniformly positive. On the one hand, a growing body of research suggests that AI use can foster student creativity in several ways. First, AI use may enhance creativity by exposing students to diverse knowledge and perspectives that stimulate new ideas. Prior research shows that informational diversity enables individuals to connect previously unrelated knowledge domains, which in turn facilitates the generation of novel ideas (Baer, 2010; Singh and Fleming, 2010). In this regard, AI tools represent a particularly powerful source of such diversity. Trained on large-scale cross-domain data, these tools provide varied perspectives and examples that help students frame problems and explore multiple solutions (Dai et al., 2023). Second, AI creates an information-rich learning environment that supports the integration of new and existing knowledge and the restructuring of knowledge frameworks, ultimately enhancing creative performance (Jarrahi, 2019; Zahedi and Song, 2014). Third, AI tools reduce the burden of routine and repetitive tasks, enabling students to devote more cognitive resources to deeper thinking and creative work (Wang et al., 2026).

On the other hand, AI use may also undermine creativity under certain conditions. Prior research cautions that over-reliance on AI can hinder students’ deep engagement with subject matter and reduce opportunities to exercise their own thinking, ultimately eroding their creativity (Dai et al., 2023). Mazhar and Arseven (2026) similarly suggest that excessive use or improper integration of AI tools may foster student dependency and reduce their capacity for original thought. Consistent with this concern, Abduljawad (2024) reported reduced creativity among third-year university students in Saudi Arabia using ChatGPT. Taken together, these studies suggest that AI use may hinder creativity when it encourages dependence on quick answers from AI systems at the expense of students’ active exploration and independent thinking.

Nevertheless, the negative effects noted above appear to arise primarily when AI is used excessively or uncritically. In contrast, the present study focuses on students’ learning-oriented use of AI for information search, idea development, and academic assistance. Used in this way, AI is more likely to function as a knowledge-supporting tool that provides diverse perspectives (Dai et al., 2023) and facilitates knowledge integration (Jarrahi, 2019). Accordingly, although prior research points to both enabling and constraining possibilities, we expect that, overall, AI use will be positively related to student creativity.

*H1*: AI use is positively related to student creativity.

### 2.2 AI use and knowledge-based dynamic capabilities

While KBDC has primarily been examined in organizational contexts, its theoretical core lies in the knowledge management process capabilities through which knowledge resources are acquired, combined, and dynamically reconfigured to support innovative outcomes (Kaur, 2023; Zheng et al., 2011). KBDCs can be understood as a procedural phenomenon that stems from micro-foundations (Kaur, 2023). Furthermore, KBDC is rooted in the dynamic capabilities view, and dynamic capabilities capture a collective phenomenon supported at the individual level (Kaur, 2023). This suggests that KBDC is not merely an organizational construct, but one rooted in underlying knowledge processes and individual-level foundations. On this basis, we argue that its underlying logic can be meaningfully adapted to the student level in the present study.

While Zheng et al. (2011) operationalized KBDC as knowledge acquisition, generation, and combination capabilities, the present study focuses on absorptive capability rather than generation capability in the student context. In organizational settings, knowledge generation is primarily viewed as an internal activity (e.g., research and development) through which new knowledge is created (Denford, 2013). However, in AI-enabled learning, students are typically less focused on internally generating new knowledge in the organizational sense, and more focused on absorbing and assimilating new knowledge provided by AI tools (Cohen and Levinthal, 1990; Denford, 2013). Therefore, absorptive capability is better suited to capture the core process through which students use AI to support creative outcomes. This adaptation is also consistent with broader KBDC scholarship, which highlights absorptive capability as an important element of KBDC processes (Kaur, 2023; Zheng et al., 2011).

Accordingly, we draw on prior KBDC research and define student-level KBDC as the ability to acquire, absorb, and combine knowledge resources to respond to changing learning contexts. Following Denford (2013), we argue that student-level KBDC is better understood as a form of dynamic capability rather than an operational capability. While general learning abilities mainly function as operational capabilities that support routine task completion, student-level KBDC reflects a dynamic capability through which students change and reconfigure their existing knowledge and abilities in response to AI inputs, thereby supporting creative idea development.

More specifically, student-level KBDC comprises three capability dimensions. Knowledge acquisition capability captures the ability to identify and obtain relevant and useful external knowledge (Zheng et al., 2011); knowledge absorptive capability reflects the ability to recognize, assimilate, and apply new knowledge, including the use and meaningful communication of such knowledge in learning-related interactions (Cohen and Levinthal, 1990; Zahra and George, 2002); and knowledge combination capability refers to the ability to integrate and apply knowledge from diverse sources (Zheng et al., 2011).

Rapid advances in AI have strengthened its capabilities in information retrieval, knowledge processing, and content generation, creating new opportunities to enhance students’ KBDC. In a similar vein, Wang et al. (2025), drawing on data from 684 content creators across various new media platforms, show that AI adoption in dynamic environments contributes to self-actualization by enhancing individuals’ abilities to explore, share, and exploit knowledge, suggesting that AI creates value by strengthening knowledge management processes. We argue that, first, AI use enhances students’ knowledge acquisition capability by enabling faster, more accurate, and easier access to information. Prior research suggests that knowledge acquisition is a fundamental part of both individual and organizational learning (Vătămănescu et al., 2023). AI systems provide timely and highly relevant knowledge through automated updates (Zhang et al., 2023), personalized recommendations, and optimized search algorithms, thereby improving information accessibility and retrieval efficiency (Sundaresan and Zhang, 2021). Such information enables students to rapidly access cross-disciplinary knowledge, broaden their knowledge sources, and build a strong foundation for creativity.

Second, AI use can strengthen knowledge absorptive capability by supporting the understanding and internalization of new knowledge. Vătămănescu et al. (2023) demonstrates that absorptive capacity is not a fixed or static capability, but a dynamic one that develops as new knowledge is assimilated learning. AI systems structure information, highlight key insights, and reveal relationships among concepts, thereby providing cognitive scaffolding that helps students process complex information more effectively (Neirotti et al., 2021). By facilitating knowledge sharing and knowledge flows within learning environments, AI supports students’ absorption of both explicit and tacit knowledge (Lin and Wu, 2015).

Third, AI use can foster students’ knowledge combination capability by enabling cross-domain integration and knowledge recombination. Creativity in learning contexts often emerges from the reconfiguration and recombination of existing knowledge (Jarrahi et al., 2023). AI systems support this process by connecting diverse knowledge elements through classification, retrieval, and filtering mechanisms that help break down knowledge silos (Shan et al., 2025). Moreover, AI tools can synthesize information from multiple sources to produce new concepts and solution pathways, enabling students to integrate prior knowledge with AI-generated insights and develop actionable solutions (Toth et al., 2025). Accordingly, we propose:

*H2a*: AI use is positively related to students’ knowledge acquisition capability.

*H2b:* AI use is positively related to students’ knowledge absorptive capability.

*H2c:* AI use is positively related to students’ knowledge combination capability.

### 2.3 Mediating role of knowledge-based dynamic capabilities

Creativity is grounded in prior knowledge, and the development of domain knowledge and skills is a fundamental prerequisite for creative performance (Bensalah and Mâţă, 2022). In this regard, knowledge acquisition capability is particularly important because it enables students to identify and obtain relevant external knowledge, thereby broadening the range of knowledge that students can draw on in generating creative ideas. Research shows that knowledge acquisition encourages creative thinking and complex problem solving among students (Huang, 2020) and serves as an important pathway linking knowledge creation processes to students’ creative behavior (Han and Zhao, 2025). Accordingly, greater knowledge acquisition capability should contribute to higher levels of student creativity.

Knowledge absorptive capability enables individuals to internalize new knowledge, build a richer knowledge base, and integrate external knowledge with what they already know. This process helps them identify new connections among ideas and supports knowledge recombination, ultimately facilitating the generation of creative ideas (Chen et al., 2025). Therefore, students with stronger knowledge absorptive capability are more likely to generate creative outcomes.

Knowledge combination capability helps individuals bring together knowledge from diverse sources, compare different perspectives, and identify latent relationships among ideas. This process deepens students’ understanding of problems and promotes the generation of original ideas, both of which are central to creativity (Arts and Fleming, 2018; Teodoridis et al., 2019). Thus, knowledge combination capability is expected to promote student creativity. Taken together, these arguments highlight knowledge-based dynamic capabilities (KBDC) as a key driver of student creativity.

Building on H1 and H2, and given the role of KBDC in fostering creativity, we argue that AI use enhances student creativity by fostering the development of KBDC.

Prior research shows that dynamic capabilities mediate the relationship between AI capabilities and organizational creativity (Almheiri et al., 2025), suggesting that AI use does not directly generate creative outcomes but operates through capability development. Likewise, Wang et al. (2025) show that AI contributes to content creators’ self-actualization through knowledge exploration, sharing, and exploitation, rather than through a simple direct effect. Similarly, KBDC mediate the impact of intellectual capital on innovative performance (Han and Li, 2015). Collectively, these studies highlight dynamic capabilities as a key mechanism through which resources and technologies are translated into creativity. Accordingly, in educational contexts, AI use is expected to enhance student creativity by strengthening students’ knowledge acquisition, absorptive, and combination capabilities. In other words, KBDC represent the mechanism through which AI use influences student creativity.

*H3:* KBDC mediate the relationship between AI use and student creativity.

*H3a:* Knowledge acquisition capability mediates the relationship between AI use and student creativity.

*H3b:* Knowledge absorptive capability mediates the relationship between AI use and student creativity.

*H3c:* Knowledge combination capability mediates the relationship between AI use and student creativity.

### 2.4 The moderating role of AI self-efficacy

Building on the relationships proposed in H2, we argue that AI use provides students with learning resources for accessing and processing knowledge, but whether these resources are translated into stronger KBDC depends on students’ AI self-efficacy—the belief that they can effectively use AI to accomplish learning tasks. Social cognitive theory posits that self-efficacy shapes individuals’ choice of activities, effort investment, and persistence in the face of obstacles, thereby influencing learning and behavioral outcomes (Bandura, 1982; Bandura, 1999). Accordingly, students with higher AI self-efficacy are more likely to engage more actively and persistently in AI-supported learning, adopt more effective strategies, and sustain exploration when tasks are complex or uncertain, which should amplify the capability-building effects of AI use.

Prior evidence supports this moderating logic. Self-efficacy has been shown to enhance the effectiveness with which information-seeking behaviors translate into cognitive gains and performance improvements (Brown et al., 2001). In educational settings, self-efficacy positively predicts students’ learning behaviors, including effort investment, absorption, and elaboration—behaviors that directly underpin deeper knowledge processing (Schweder, 2019). Moreover, research on knowledge management indicates that computer self-efficacy beliefs are closely related to knowledge sharing and exchange behaviors (Hsu et al., 2007), suggesting that efficacy influences not only whether individuals use digital tools but also how effectively tool use facilitates knowledge exchange and utilization.

This mechanism is particularly relevant in AI-supported learning. Student interaction with generative AI has been linked to learning achievement, with self-efficacy playing a pivotal role in the process (Liang et al., 2023). Relatedly, students’ AI use and AI literacy are closely associated with AI self-efficacy (Bewersdorff et al., 2025), highlighting AI self-efficacy as a key psychological foundation for effective AI-enabled learning and knowledge work. Therefore, when AI self-efficacy is high, students should be better able to leverage AI use to support (a) more proactive and efficient knowledge acquisition, (b) deeper assimilation and internalization of new knowledge, and (c) more effective recombination and integration of knowledge across sources, thereby strengthening the positive relationship between AI use and the three dimensions of KBDC. Accordingly, we propose:

*H4:* AI self-efficacy positively moderates the relationship between AI use and KBDC.

*H4a:* AI self-efficacy positively moderates the relationship between AI use and knowledge acquisition capability.

*H4b:* AI self-efficacy positively moderates the relationship between AI use and knowledge absorptive capability.

*H4c:* AI self-efficacy positively moderates the relationship between AI use and knowledge combination capability.

### 2.5 The moderating role of AI trust

Building on H2, which proposes that AI use enhances students’ KBDC, we further argue that this relationship depends on students’ trust in AI systems. Following prior trust research, we define AI trust as students’ positive beliefs that AI systems are useful, reliable, and operate as intended (Lockey et al., 2021). Prior research suggests that AI trust is central to human–AI interaction because it shapes users’ acceptance of AI technologies and their reliance on AI or automated systems (Glikson and Woolley, 2020; Hoff and Bashir, 2015).

Although trust is necessary for effective AI use, higher AI trust may also shape how students engage with and process AI-generated information. Recent studies in higher education and generative AI contexts have treated AI trust as an important moderating factor that shapes how AI-related experiences translate into educational outcomes (Chiu, 2025; Shahzad et al., 2025). More directly, Cui et al. (2025) show that learners’ trust in AI is positively associated with their willingness to accept AI suggestions and their receptivity to system-generated recommendations. At the same time, they highlight the need for learners to maintain autonomy and critical judgment when using AI tools (Cui et al., 2025). Related evidence further shows that AI trust can amplify the negative relationship between AI use and users’ critical thinking (Kulal, 2025). Thus, when students hold stronger positive beliefs about AI’s reliability and usefulness, they may be more likely to treat AI-generated outputs as valid learning inputs and perceive less need to verify and critically evaluate such information before using it in their own learning.

This matters for KBDC because its three dimensions require students to move beyond simply accepting external knowledge inputs and to actively engage with knowledge by evaluating, internalizing, and recombining it for learning and creative outcomes (Zahra and George, 2002; Zheng et al., 2011). Therefore, when students with higher AI trust accept AI-generated outputs without sufficient verification or critical engagement, AI use may be less strongly associated with the development of knowledge acquisition, absorptive, and combination capabilities. Accordingly, we propose:

*H5:* AI trust negatively moderates the relationship between AI use and KBDC.

*H5a:* AI trust negatively moderates the relationship between AI use and knowledge acquisition capability.

*H5b:* AI trust negatively moderates the relationship between AI use and knowledge absorptive capability.

*H5c:* AI trust negatively moderates the relationship between AI use and knowledge combination capability.

**Figure 1:**
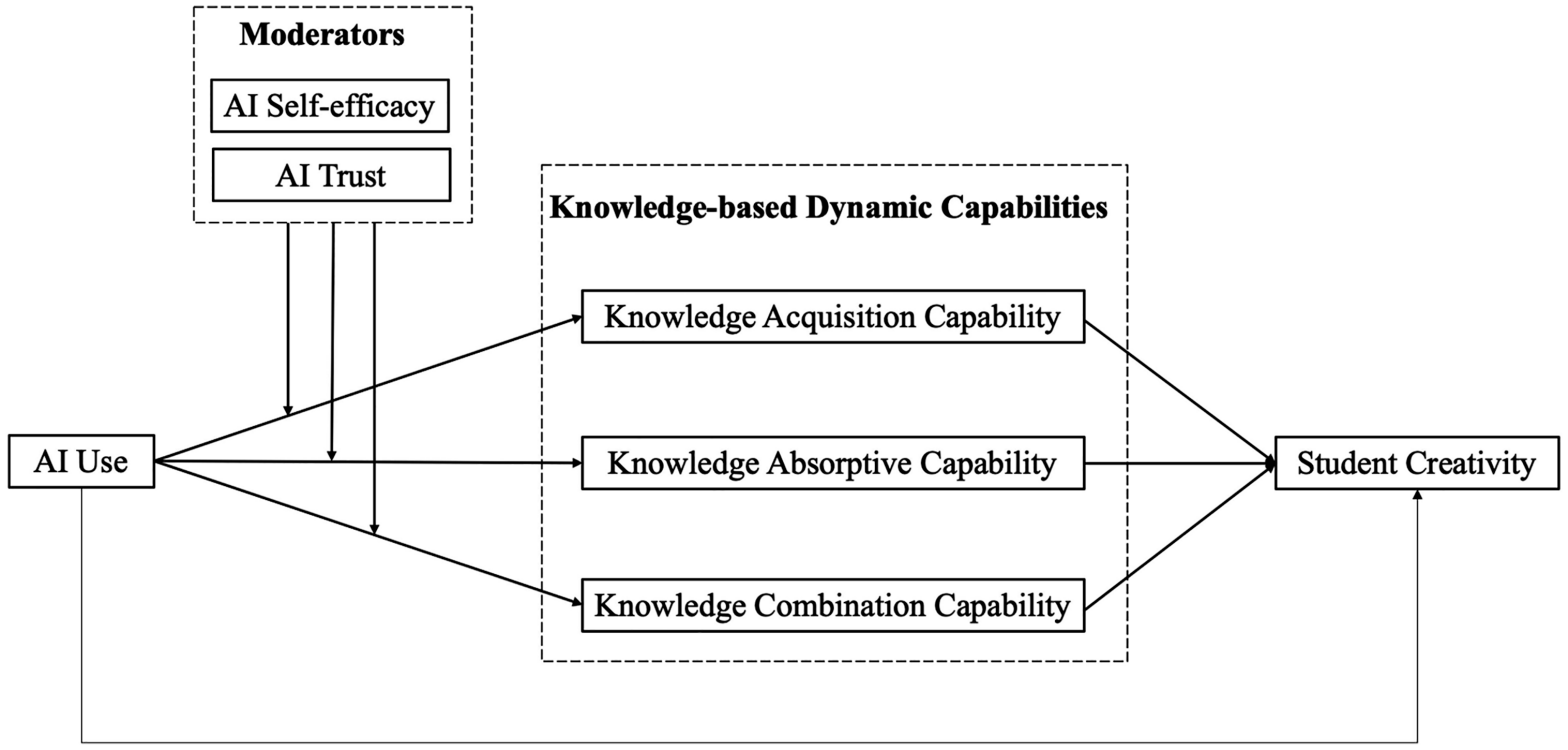
Hypothetical model. AI, artificial intelligence.

Figure 1 presents the conceptual model of this study. Drawing on the KBDC perspective, the model specifies AI use as the independent variable and student creativity as the dependent variable. Knowledge acquisition capability, knowledge absorptive capability, and knowledge combination capability are proposed as parallel mediators linking AI use to student creativity, while AI self-efficacy and AI trust are positioned as first-stage moderators of the relationships between AI use and these three capability dimensions. Together, these relationships explain both how AI use influences student creativity and when its effects on capability development are likely to be strengthened or weakened.

## 3 Methods

### 3.1 Sample and data sources

In this study, a questionnaire was used to collect data. Before any data collection began, we obtained ethical approval for this study from the ethics committee of our university (Approval No. CDUTBS-EC-2025-166). Before the formal survey, in order to improve the reliability, validity and clarity of the questionnaire, a small sample of college students in China was pre-surveyed. A total of 120 questionnaires were distributed, and 98 valid questionnaires were recovered, with an effective recovery rate of 81.67%. Combined with the analysis results of the pre-survey data, as well as the constructive opinions put forward by academic experts and interviewees, some items of the first version of the questionnaire were revised, and at the same time, the wording and layout of the remaining items were refined and optimized to improve the quality of the scale.

This study adopted a sampling strategy that combined stratified sampling with convenience sampling to enhance sample representativeness and coverage while maintaining the feasibility of data collection (Hu et al., 2025; Ruffino et al., 2025). The target population of this study was university students. First, based on the accessibility of potential participants, we selected three universities in Sichuan Province, China, covering three institutional types: comprehensive universities, science and engineering oriented universities, and teacher education-oriented universities. Second, considering that students from different disciplines and years of study may differ in their AI use and creativity, we used discipline and year of study as stratification variables (Shahzad et al., 2025; Zhou and Peng, 2025). Students from science, engineering, management, and other humanities and social science disciplines, as well as from different years of study, were included. Subject to voluntary participation, two to three classes from different majors were randomly selected for the survey. This sampling procedure was intended to capture variation in students’ AI use, AI self-efficacy, AI trust, KBDC, and creativity across different stages of university study.

During the data collection stage, the questionnaires were distributed in class and through course-related communication groups. Before completing the survey, participants read the participant information sheet and consent form, which explained the purpose of our study, the voluntary nature of their participation, and the use of the research data. Teachers explained the purpose, duration, and value of the survey to students, emphasizing voluntary participation and information confidentiality. All participants provided informed consent before proceeding with the survey. The questionnaire was completed anonymously, and students were clearly informed that their personal information would not be disclosed. We treated all responses confidentially and anonymized the data before conducting data analysis.

This study carried out two data collections in November 2025 and May 2026, respectively. After analyzing the variance of the sample data in the two stages, it was found that there were no significant differences, so the analysis can be combined (Yi et al., 2022). A total of 982 questionnaires were distributed in the survey. After strictly excluding invalid questionnaires such as incomplete answers, regular answers, and short answering time, 764 questionnaires were finally valid, with an effective recovery rate of 77.8%. As shown in Table 1, the demographic characteristics of the final sample are as follows: in terms of gender, 389 (50.92%) men and 375 (49.08%) women; Grade distribution, freshman 362 (47.38%), sophomore 119 (15.58%), junior 171 (22.38%), senior 112 (14.66%); Disciplines, science 163 (21.34%), engineering 258 (33.77%), management 200 (26.18%), and other humanities and social sciences 143 (18.72%). The interviewed college students include different genders, different grades and different disciplines, making the research conclusions more universal and valuable.

**Table 1:** Basic information of survey participants.

Variable	Category	Number	Proportion(%)	Cumulative percentage (%)
Gender	Male	389	50.92%	50.92%
	Female	375	49.08%	100%
Age	Under 18	34	4.45%	4.45%
	18–22 years old	699	91.49%	95.94%
	23–25 years old	31	4.06%	100%
Grade	Freshman	362	47.38%	47.38%
	Sophomore	119	15.58%	62.96%
	Junior	171	22.38%	85.34
	Senior	112	14.66%	100%
Discipline	Science	163	21.34%	21.34%
	Engineering	258	33.77%	55.11%
	Management	200	26.18%	81.29%
	Other humanities and social sciences	143	18.72%	100%

Following Podsakoff et al. (2003), Chun et al. (2025), and Kock (2017), this study employed two methods to assess common method bias: Harman’s single-factor test and the full collinearity test. As shown in Table 2, Harman’s one-factor test showed that the first factor explained only 38.53% of the variance, below the threshold of 40%. The results of the complete collinearity test indicate that all structural variance factors are below an acceptable cutoff value 5. Therefore, it can be inferred that the common method bias does not constitute a major problem in this study.

**Table 2:** Reliability and validity analysis.

Variable	Item	Standardized factor loadings	α value	CR	AVE	VIF
Student creativity	CR1	0.841	0.903	0.917	0.933	2.889
	CR2	0.901				3.428
	CR3	0.828				2.720
	CR4	0.854				2.814
AI use	USE1	0.655	0.754	0769	0.528	1.600
	USE2	0.722				1.678
	USE3	0.796				1.854
KAC	KAC 1	0.778	0.793	0.849	0.652	1.757
	KAC 2	0.822				2.179
	KAC 3	0.821				2.243
KAbC	KAbC 1	0.900	0.908	0.928	0.723	3.933
	KAbC 2	0.916				4.525
	KAbC 3	0.884				3.508
	KAbC 4	0.829				2.916
	KAbC 5	0.704				2.099
KCC	KCC 1	0.864	0.914	0.928	0.810	3.411
	KCC 2	0.937				4.646
	KCC 3	0.898				3.673
AI self-efficacy	XN1	0.816	0.828	0.872	0.694	1.888
	XN2	0.839				2.227
	XN3	0.844				2.292
AI trust	XR1	0.673	0.724	0.791	0.509	1.343
	XR2	0.715				1.628
	XR3	0.749				1.480
	XR4	0.651				1.707

### 3.2 Measures

The variables that need to be measured in the study mainly include student creativity, Al use, KBDC (specifically including KAC, KAbC, KCC), AI self-efficacy, and Al trust. This study finally chooses to use the special 7-point scoring method. During the measurement process, the numbers “1–7” indicate the degree of approval of the surveyed object on a certain item, “1” means very inconsistent, “7” means very consistent, and “4” means neutral and generally consistent. The specific measurement items for each variable are presented in Table 3.

**Table 3:** Scale items and description.

Scale items	*M*	SD
Student creativity		
I will take the lead in trying new ideas or new methods.	4.79	1.124
I will actively explore new ideas and approaches to solve problems.	4.93	1.066
I will come up with innovative ideas that are relevant to my field of study.	4.67	1.130
I like to use all kinds of resources creatively.	4.92	1.082
Al use		
I used AI to assist in many of my decision-making activities.	4.37	1.526
I used AI to assist in collecting a large amount of materials.	5.12	1.256
I used AI to assist in completing many of my study or work tasks.	4.82	1.307
KAC		
I can acquire more knowledge from school teaching.	5.02	1.122
I can acquire more knowledge from social practice.	5.28	0.997
I will take the initiative to get more knowledge from other ways.	5.24	1.017
KAbC		
I can quickly access data and information about the task.	5.01	1.017
I can quickly understand the information collected or transmitted by others.	5.07	0.990
I can quickly identify valuable information.	5.05	1.015
I can quickly and completely learn the knowledge I lack.	4.82	1.091
I can pass on the new knowledge to other students in time.	4.76	1.138
KCC		
I can effectively integrate knowledge from different professional fields.	4.74	1.090
I am able to integrate new knowledge effectively.	4.95	1.022
I can effectively integrate newly acquired knowledge with existing knowledge.	4.98	1.016
AI self-efficacy		
When faced with difficult tasks, I am sure that I can do it by using AI.	4.54	1.165
I believe I can easily use different AI tools to complete tasks.	4.69	1.116
I can achieve most of the goals I set by using AI.	4.77	1.064
AI trust		
I believe that AI will prioritize users’ interests over its own.	4.20	1.316
I think AI excels in assistant roles.	4.96	1.173
I believe that AI can reliably deliver the outcomes I expect as long as appropriate prompts are input.	4.39	1.266
I think AI can help people study or work more efficiently.	5.42	1.005

#### 3.2.1 Student creativity

Following previous studies (Fan and Cai, 2022; Farmer et al., 2003), student creativity was measured using a four-item scale. Sample items are presented as follows: “I will take the lead in trying new ideas or new methods” and “I will actively explore new ideas and approaches to solve problems.”

#### 3.2.2 Al use

In line with prior research (Man Tang et al., 2022; Yam et al., 2023), this study measured Al use using an established scale. The items are listed as follows: “I used AI to assist in many of my decision-making activities,” “I used AI to assist in collecting a large amount of materials,” and “I used AI to assist in completing many of my study or work tasks.”

#### 3.2.3 KBDC

The scales used to measure the three dimensions of student-level KBDC were adapted from established measures of organizational- and individual-level knowledge capability scales (Zhang et al., 2018; Zheng et al., 2010) and were guided by prior research on KBDC and absorptive capacity (Zahra and George, 2002). More specifically, the KAC and KCC items were adapted primarily from Zheng et al. (2010) organizational-level KBDC scale, which draws on prior work such as Lane et al. (2001) and Prieto and Easterby-Smith (2006). For KAC, we adapted Zheng et al. (2010) organizational-level KAC items to reflect students’ acquisition of knowledge from school teaching, social practice, and other learning channels. Representative items include “I can acquire more knowledge from school teaching” and “I can acquire more knowledge from social practice.” These items are consistent with our definition of student-level KAC as the ability to identify and obtain relevant and useful external knowledge.

For KCC, Zheng et al. (2010) items concerning the integration of internal and external knowledge, cross-domain knowledge, and newly acquired knowledge were adapted to assess students’ ability to integrate knowledge across professional fields and combine newly acquired knowledge with existing knowledge. Representative items include “I can effectively integrate newly acquired knowledge with existing knowledge” and “I can effectively integrate knowledge from different professional fields.” These items align with our definition of student-level KCC, which emphasizes students’ ability to integrate and apply knowledge from diverse sources.

The items for knowledge absorptive capability (KAbC) were adapted from Zhang et al. (2018) individual KAbC scale and grounded in Zahra and George (2002) conceptualization of absorptive capacity. Because the original items were developed in organizational and work-related contexts, we reviewed and adapted them to the student learning context. We retained five items that capture students’ KAbC, including their ability to access task-related information, identify valuable information, understand information obtained from others, learn the knowledge they lack, and communicate newly acquired knowledge to other students. These items correspond to our definition of KAbC as students’ ability to recognize, assimilate, and apply new knowledge, including the use and meaningful communication of such knowledge in learning-related interactions. Representative items include “I can quickly understand the information collected or transmitted by others” and “I can quickly identify valuable information.” The item “I can pass on the new knowledge to other students in time” was retained because, following Zahra and George (2002), it captures the knowledge-exploitation aspect of absorptive capability in the student learning context. The final KBDC scale consisted of 11 items: three items for KAC, five items for KAbC, and three items for KCC (Table 3).

#### 3.2.4 Al self-efficacy

This study employed a three-item scale adapted from Khansa et al. (2017) and Shao et al. (2022), to measure AI self-efficacy. The original instrument was designed to assess Al self-efficacy and is well suited to the present research context. Several adjustments were made to fit the current research setting. Sample items are presented as follows: “I believe I can easily use different AI tools to complete tasks.” and “I can achieve most of the goals I set by using AI.”

#### 3.2.5 Al trust

The measurement scale for AI trust was mainly informed by the research of Chowdhury et al. (2022), and Kong et al. (2023), consisting of four items in total. Representative items are as follows: “I believe that AI will prioritize users’ interests over its own.”; “I believe that AI can reliably deliver the outcomes I expect as long as appropriate prompts are input.”

### 3.3 Regression model setting

In order to test each hypothesis, this study constructed the following regression model:

First, in order to test the direct impact of AI use on student creativity, the model was set up as follows:

$$Creativity=\alpha_{0}+\alpha_{1}AIuse+\sum_{\gamma k}Controls+\varepsilon_{1}$$

Among them, Creativity represents student creativity, Controls represents control variables (including gender, age, grade, subject category), *k* is the serial number subscript of control variables, representing the kth control variable, and *ε* is random error term. Secondly, in order to test the impact of AI use on each dimension of knowledge-based dynamic capabilities, with knowledge-based dynamic capabilities as the dependent variable, the model was set as follows:

$$KBDC=\beta_{0}+\beta_{1}AIuse+\sum_{\gamma 1k}Controls_{k}+\varepsilon_{2}$$

Among them, KBDC represents knowledge-based dynamic capabilities (including KAC, KAbC and KCC). Thirdly, in order to test the mediating effect of AI self-efficacy, the independent variable and three mediating variables were included in the regression model of creativity at the same time:

$$\begin{array}{l} Creativity=\alpha_{2}+\alpha_{3}AIuse+\lambda_{1}KAC+\lambda_{2}KAbC\\ \;\;\;\;\;\;\;\;\;\;\;\;\;\;\;\;\;\;\;\;\;\;\;\;\;+\lambda_{3}KCC+\sum_{\gamma 2k}Controls_{k}+\varepsilon_{3} \end{array}$$

Finally, in order to test the moderating effect of AI self-efficacy (AISE) and AI trust (AIT) on AI use and AI self-efficacy, taking KBDC as the dependent variable as an example, the model was set as follows:

$$\begin{array}{l} KBDC=\beta_{2}+\beta_{3}AIuse+\beta_{4}AISE+\beta_{5}AIT+\beta_{6}\left(AIuse\times AISE\right)\\ \;\;\;\;\;\;\;\;\;\;\;\;\;\;\;\;\;+\beta_{7}\left(AIuse\times AIT\right)+\sum_{\gamma 3k}Controls_{k}+\varepsilon_{4} \end{array}$$

The results of regression analysis are presented in the next section.

## 4 Results

This section presents the empirical results following a standardized analytical procedure. All data processing and statistical analyses were conducted using SPSS 24.0 and AMOS to improve the reproducibility of the results. The analytical workflow strictly follows the canonical empirical research paradigm: we first conducted measurement model validation including reliability, convergent validity, and discriminant validity tests to ensure the scientific and stability of the questionnaire data. Next, confirmatory factor analysis was performed to verify the optimal fitting structure of the research model. Subsequently, descriptive statistics and correlation analysis were implemented to test data normality and provide preliminary empirical evidence. Finally, hierarchical regression and Bootstrap method were adopted to systematically examine the research hypotheses. All analytical steps are reported in detail below to clarify the derivation logic of all empirical results and guarantee research replicability.

### 4.1 Reliability and validity analysis

This study used SPSS 24.0 and AMOS software to test the reliability and validity of the questionnaire items, and the results are shown in Table 2. Specifically, all Cronbach’s alpha coefficients were greater than 0.7, indicating high reliability and consistency of the questionnaire. The combined reliability (CR) value of each variable was greater than 0.7. The mean variance extraction (AVE) values of all variables were greater than 0.5, and the factor loads of each scale item of all variables were higher than 0.6. Therefore, the variables used in this study still have good combination reliability and convergent validity.

In order to further test the discriminative validity between the variables, this study uses the heterotrait-monotrait ratio (HTMT) criterion proposed by Henseler et al. (2015) for evaluation. The results are shown in Table 4, with HTMT values between all variables below the strict threshold of 0.85, indicating good discriminative validity between the latent variables.

**Table 4:** The results of HTMT.

Variables	1	2	3	4	5	6
1. Student creativity						
2. AI use	0.148					
3. KAC	0.632	0.213				
4. KAbC	0.660	0.193	0.831			
5. KCC	0.649	0.208	0.713	0.815		
6. AI self-efficacy	0.533	0.470	0.521	0.584	0.552	
7. AI trust	0.260	0.555	0.300	0.291	0.277	0.475

### 4.2 Confirmatory factor analysis

This paper conducts a confirmatory factor analysis on students’ creativity, AI use, KAC, KAbC, KCC, AI self-efficacy and AI trust, and the results are shown in Table 5. The CMIN/DF value of the seven-factor model is 3.247, which is lower than the critical value of 5; The RMSEA value was 0.056, lower than the critical value of 0.08; TLI (0.940), CFI (0.948) and GFI (0.911) were all above the benchmark of 0.9. As shown in Table 5, the seven-factor model produced the best fit index compared with the other six alternative models, and also verified good discriminative validity among the variables.

**Table 5:** Confirmatory factor analysis.

Model	CMIN/DF	RMSEA	GFI	CFI	TLI
Seven-factor model	3.247	0.056	0.911	0.948	0.940
Six-factor model	4.451	0.069	0.864	0.918	0.907
Five-factor model	6.074	0.084	0.820	0.878	0.864
Four-factor model	9.324	0.107	0.733	0.797	0.777
Three-factor model	11.234	0.131	0.635	0.749	0.725
Two-factor model	13.391	0.135	0.609	0.695	0.667
One-factor model	14.554	0.137	0617	0.665	0.636

### 4.3 Descriptive statistics and correlation analysis

The results of descriptive statistics and correlation analysis are shown in Table 6. It can be seen from Table 6 that the absolute values of skewness and kurtosis for all continuous variables are less than 3 and 7 (Wei et al., 2022), respectively, indicating that the sample data follow a normal distribution. It can also be seen from Table 6 that AI use is significantly positively associated with student creativity, KBDC is significantly positively associated with student creativity, and AI use is significantly positively associated with KBDC. The correlation signs are broadly consistent with theoretical predictions, providing preliminary statistical support for subsequent regression analysis and hypothesis testing.

**Table 6:** Descriptive statistics and correlation analysis of main variables.

Variables	Mean	SD	Skewness	Kurtosis	1	2	3	4	5	6
1. Student creativity	4.764	1.002	0.349	−0.025	1					
2. AI use	4.772	1.120	−0.123	0.318	0.117*	1				
3. KAC	5.179	0.880	0.123	0.137	0.522**	0.162**	1			
4. KAbC	4.943	0.898	0.395	−0.035	0.602**	0.158**	0.704**	1		
5. KCC	4.892	0.963	0.241	−0.052	0.587**	0.172**	0.606**	0.817**	1	
6. AI self-efficacy	4.665	0.962	0.168	0.203	0.439**	0.373**	0.422**	0.507**	0.479**	1
7. AI trust	4.744	0.885	0.094	−0.039	0.181**	0.405**	0.218**	0.230**	0.219**	0.365**

*P < 0.05, **P < 0.01.

### 4.4 Hypothesis testing

#### 4.4.1 Testing direct and mediating effects

**Figure 2:**
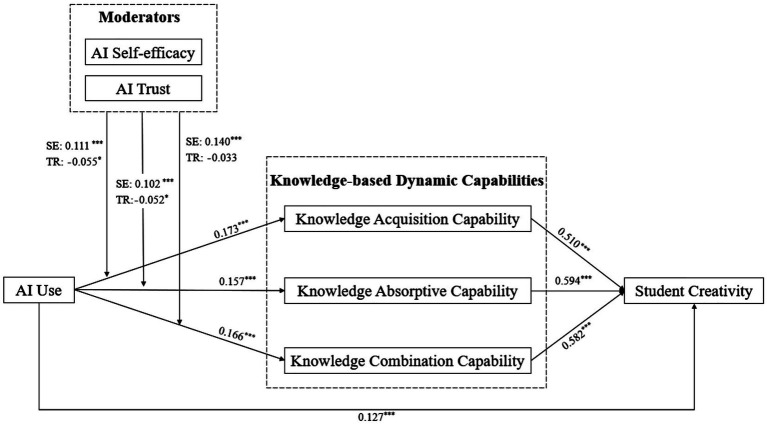
Results of the proposed research model linking AI use and Student creativity. AI, artificial intelligence; SE, AI self-efficacy; TR, AI trust. For each vertical arrow, the upper coefficient denotes the moderating effect of AI self-efficacy, whereas the lower coefficient denotes that of AI trust. *p < 0.1, **p < 0.05, ***p < 0.01.

To examine the direct and mediating effects proposed in this study, we employed hierarchical regression analysis. This approach involves adding independent variables in successive steps to determine whether the incremental contribution of newly introduced variables to the model’s explanatory power is statistically significant. If a variable continues to demonstrate a meaningful contribution after being entered stepwise, we can interpret it as having a unique role that cannot be replaced by other variables (Zhang et al., 2025). The data in this study satisfy the applicable conditions for hierarchical regression: the dependent variable is continuous; there are no fewer than two independent variables; a linear relationship exists between the independent and dependent variables (see Table 6); no severe multicollinearity is present (all VIF values are below 5); and we removed significant outliers. Accordingly, we used hierarchical regression analysis to test the main effect of AI use on student creativity, the mediating effect of AI self-efficacy, and the moderating effects of AI self-efficacy and AI trust on the relationship between AI use and knowledge-based dynamic capabilities. Figure 2 presents the results.

First, as shown in Model 2 in Table 7, after controlling for gender, age, grade, and subject category, AI use had a significant positive effect on student creativity (*β* = 0.127, *p* < 0.01), supporting hypothesis H1. This suggests that students’ use of AI as an instrument can help boost their creativity. In addition, the results of Models 3, 4, and 5 show that AI use has a positive and significant effect on KAC (Model 3, *β* = 0.173; *p* < 0.01), KAbC (Model 4, *β* = 0.157; *p* < 0.01), and KCC (Model 5, *β* = 0.166; *p* < 0.01). This shows that AI use has a significant positive impact on KAC, KAbC, and KCC, thus verifying the hypotheses H2a, H2b, and H2c.

**Table 7:** Results of direct and mediating effect tests.

Variables	Student creativity		KAC	KAbC	KCC
	Model 1	Model 2	Model 3	Model 4	Model 5
AI use		0.127*** (3.363)	0.173*** (4.600)	0.157*** (4.173)	0.166*** (4.416)
Gender	0.106*** (2.861)	0.105*** (2.859)	0.058 (1.576)	0.059 (1.597)	0.049 (1.325)
Age	0.051 (1.37)	0.053 (1.449)	0.015 (0.416)	0.013 (0.358)	−0.006 (−0.164)
Grade	−0.049 (−1.067)	−0.075 (−1.621)	−0.076 (−1.634)	−0.045 (−0.973)	0.006 (0.138)
Discipline	0.036 (0.776)	0.033 (0.717)	0.033 (0.732)	0.067 (1.468)	0.020 (0.434)
*F*	2.743**	4.487***	4.946***	4.706***	4.839***
R-squared	0.015	0.030	0.033	0.032	0.033
Adjusted R-squared	0.010	0.024	0.027	0.025	0.026

*P < 0.1, **P < 0.05, ***P < 0.01.

Table 8 further reveals the mediating role of KBDC in the relationship between AI use and student creativity. Models 1 to 3 introduce KAC, KAbC, and KCC as mediating variables, respectively. The results show that KAC has a significant positive impact on student creativity (*β* = 0.510, *p* < 0.01), KAbC has a significant positive impact on student creativity (*β* = 0.594, *p* < 0.01), and KCC has a significant positive impact on student creativity (*β* = 0.582, *p* < 0.01). At the same time, after including these three mediating variables, the direct effect of AI use on student creativity is not significant. This shows that KAC, KAbC, and KCC play a completely mediating role between the use of AI and student creativity. Therefore, it is assumed that H3a, H3b and H3c are supported. Taken together, these findings provide support for the hypothesis H3, suggesting that AI use indirectly enhances student creativity by promoting KBDC. In Model 4, all mediating variables were included simultaneously. The mediating effects remained statistically significant, which further verified Hypothesis H3.

**Table 8:** Results of mediating effect tests.

Variables	Student creativity			
	Model 1	Model 2	Model 3	Model 4
AI use	0.038 (1.165)	0.033 (1.088)	0.030 (0.965)	0.012 (0.394)
KAC	0.510*** (15.846)			0.172*** (4.223)
KAbC		0.594*** (19.742)		0.251*** (4.485)
KCC			0.582*** (19.121)	0.276*** (5.523)
Gender	0.076** (2.383)	0.070** (2.368)	0.077** (2.562)	0.067** (2.337)
Age	0.046 (1.436)	0.045 (1.535)	0.057* (1.896)	0.049* (1.711)
Grade	−0.037 (−0.914)	−0.048 (−1.295)	−0.079** (−2.089)	−0.053 (−1.453)
Discipline	0.016 (0.400)	−0.007 (−0.191)	0.021 (0.571)	0.055 (0.134)
*F*	46.987***	70.721***	66.571***	63.254***
R-squared	0.282	0.372	0.358	0.414
Adjusted R-squared	0.276	0.367	0.352	0.408

*P < 0.1, **P < 0.05, ***P < 0.01.

In addition, we adopted the Bootstrap method to test the mediating effects. As shown in Table 9, when KAC, KAbC, and KCC are introduced into the model as mediating variables, the direct effect between AI use and student creativity is not significant, while the 95% confidence interval of the indirect effect of the three mediating variables does not contain 0, indicating that the three dimensions of KBDC play a complete mediating role between AI use and student creativity. Specifically, KCC had the largest indirect effect (0.041) and KAC had the smallest indirect effect (0.027). The mediating effects of KAC, KAbC, and KCC accounted for 26.2, 34 and 39.8% of the total effects, respectively.

**Table 9:** Bootstrap mediation effect tests.

Path	Effect value	SE	LLCI	ULCI	Effect size
Direct effect					
AI use→ Student creativity	0.011	0.027	−0.042	0.063	
Indirect effect					
AI use→ KAC → Student creativity	0.027	0.010	0.010	0.048	26.2%
AI use→ KAbC→ Student creativity	0.035	0.013	0.013	0.066	34.0%
AI use→ KCC → Student creativity	0.041	0.014	0.017	0.071	39.8%
Total indirect effect	0.103	0.024	0.058	0.151	100%
Total effect	0.113	0.034	0.047	0.180	

#### 4.4.2 Moderation effect tests

Table 10 reports the moderating effect of AI use on KBDC. The interaction between AI use and AI self-efficacy is significantly positively associated with KAC (*β* = 0.111; *p* < 0.01), KAbC (*β* = 0.102; *p* < 0.01), and KCC (*β* = 0.140; *p* < 0.01). Therefore, it is assumed that H4a, H4b, and H4c are supported. Overall, these findings provide support for the hypothesis H4, suggesting that the stronger students’ confidence in their ability to use AI, the stronger the positive impact of AI use on their KBDC.

**Table 10:** Results of moderation effect tests.

Model and variables	KAC	KAbC	KCC
	Model 1	Model 2	Model 3
AI use *AI self-efficacy	0.111*** (3.193)	0.102*** (3.065)	0.140*** (4.142)
AI use *AI trust	−0.055* (−1.786)	−0.052* (−1.839)	−0.033 (−0.981)
AI self-efficacy	0.414*** (11.015)	0.512*** (14.322)	0.477*** (13.101)
AI trust	0.062 (1.614)	0.057 (1.556)	0.044 (1.189)
Al use	−0.005 (−0.130)	−0.054 (−1.457)	−0.033 (−0.864)
Gender	0.025 (0.729)	0.021 (0.647)	0.010 (0.301)
Age	0.007 (0.193)	0.007 (0.213)	−0.016 (−0.478)
Grade	−0.089** (−2.115)	−0.063 (−1.559)	−0.011 (−0.272)
Discipline	0.032 (0.778)	0.064 (1.604)	0.017 (0.426)
*F*	20.067***	30.296***	26.560***
R-squared	0.202	0.276	0.251
Adjusted R-squared	0.192	0.267	0.241

*P < 0.1, **P < 0.05, ***P < 0.01.

**Figure 3:**
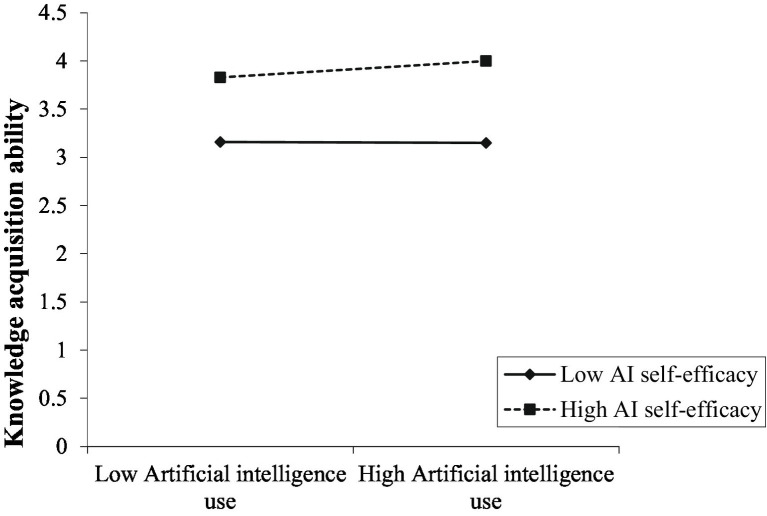
Moderating effect of Al self-efficacy on the relationship between AI use and KAC.

**Figure 4:**
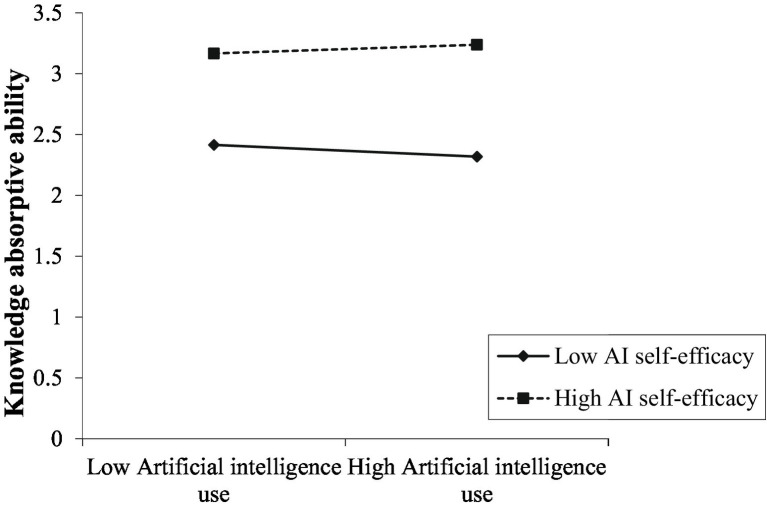
The moderating effect of Al self-efficacy on the relationship between AI use and KAbC.

**Figure 5:**
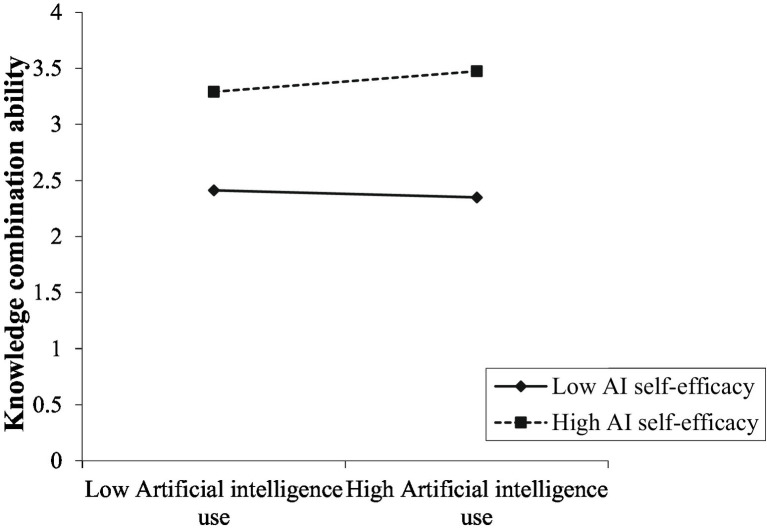
The moderating effect of AI self-efficacy on the relationship between AI use and KCC.

**Figure 6:**
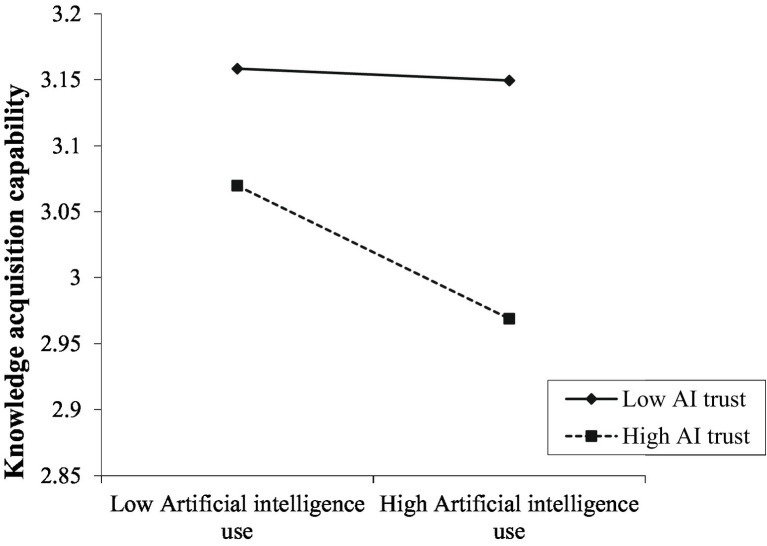
The moderating role of AI trust in the relationship between AI use and KAC.

**Figure 7:**
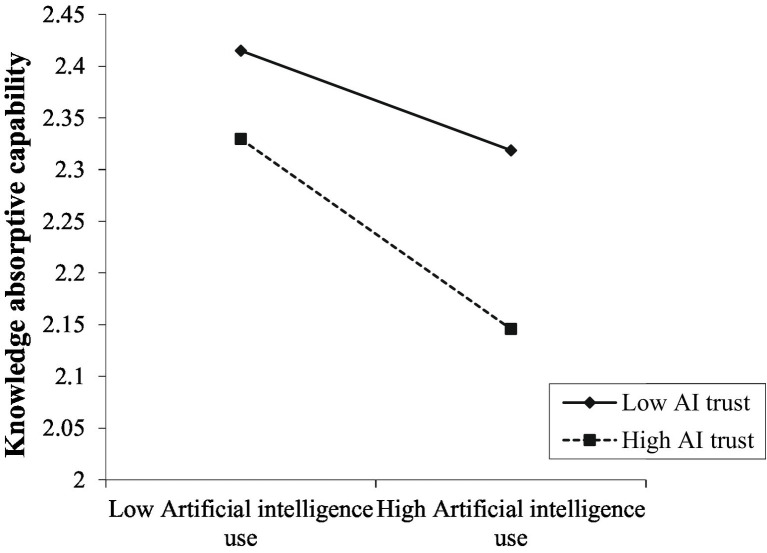
The moderating effect of AI trust on the relationship between AI use and KabC.

To visually illustrate these moderating effects, this study constructed the moderating effect diagrams, as presented in Figures 3–5. The results show that AI self-efficacy has a significant positive moderating effect, that is, high AI self-efficacy enhances the positive impact of Al use on KAC, KAbC and KCC. In addition, Table 10 reveals that AI trust weakens the positive relationship between AI use and KAC and KAbC. Specifically, the interaction of AI use and AI trust has a negative and significant effect on KAC (*β* = −0.055, *p* < 0.1) and KAbC (*β* = −0.052, *p* < 0.1), which supports Hypotheses H5a and H5b. However, the interaction between AI use and AI trust is negative but not significant on KCC (*β* = −0.033, *p* > 0.1), indicating that Hypothesis H5c is not supported. Figures 6,7 visually demonstrate the moderating role of AI trust. As shown in the figure, higher AI trust will weaken the positive impact of AI use on KAC and KAbC.

### 4.5 Robustness and endogeneity checks

This study used two additional approaches to examine the robustness of the findings and to address potential endogeneity concerns. First, we added additional learning-related control variables, namely, exploitative learning and exploratory learning, in research model 2 (see Table 11). In educational settings, exploitative learning and exploratory learning are widely recognized as important learning behaviors through which individuals develop creativity (Lennerts et al., 2020; Liu Z. et al., 2026). These two constructs capture students’ learning-related tendencies. Specifically, exploitative learning focuses on refining and improving existing knowledge and skills, whereas exploratory learning involves seeking and acquiring new knowledge and skills to address emerging problems and stimulate creative thinking.

**Table 11:** Robustness and endogeneity checks.

Model and variables	Student creativity		AI use
	Model 1	Model 2	Model 3
AI use		0.026** (2.399)	
Student creativity			−0.038 (−0.719)
KAC			0.076 (1.450)
KAbC			−0.036 (−0.508)
KCC			0.069 (1.070)
Exploitative learning	0.147*** (3.603)	0.148*** (3.613)	0.078 (1.370)
Exploratory learning	0.578*** (14.135)	0.579*** (14.113)	0.080 (1.213)
Gender	0.050* (1.855)	0.049* (1.851)	−0.006 (−0.175)
Age	0.020 (0.733)	0.019 (0.724)	−0.023 (−0.653)
Grade	−0.030 (−0.908)	−0.028 (−0.840)	0.207*** (4.635)
Discipline	0.027 (0.826)	0.027 (0.830)	0.020 (0.442)
*F*	100.400***	117.265***	7.053
R-squared	0.495	0.495	0.090
Adjusted R-squared	0.490	0.491	0.077

*P < 0.1, **P < 0.05, ***P < 0.01.

Consistent with prior research (Ge et al., 2016; Lin et al., 2015), exploitative and exploratory learning were measured using a validated eight-item scale. Four items were used to measure exploitative learning, with representative items including “In my studies, I focus on consolidating existing knowledge and skills in familiar fields” and “I often adjust my methods to improve learning efficiency.” The remaining four items measured exploratory learning, with representative items including “In my studies, I acquire entirely new knowledge and skills to solve problems” and “I often develop interest in knowledge and skills in unknown fields.” As shown in Model 2 of Table 11, AI use remained positively and significantly associated with student creativity after additionally controlling for exploitative and exploratory learning (*β* = 0.026, *p* < 0.05). This result provides further support for the robustness of the main finding.

Second, we conducted a reverse causality check. It is possible that students with higher creativity or stronger KBDC may be more likely to use AI. To examine this possibility, we estimated an alternative model in which AI use was treated as the dependent variable, while student creativity and the three KBDC dimensions were entered as predictors. As shown in Model 3 of Table 11, student creativity, knowledge acquisition capability, knowledge absorptive capability, and knowledge combination capability did not significantly predict AI use (*β* = −0.038, *p* > 0.1; *β* = 0.076, *p* > 0.1; *β* = −0.036, *p* > 0.1; *β* = 0.069, *p* > 0.1, respectively). These results do not fully rule out endogeneity, but they suggest that the reverse-causality explanation is not strongly supported by our data.

## 5 Discussion

### 5.1 Research findings

Drawing on the KBDC theory, this study examines how AI use influences student creativity, while also considering the moderating roles of AI self-efficacy and AI trust. Using survey data from 764 university students in China and applying hierarchical regression analysis, the study yields the following results. First, AI use is positively associated with student creativity. Second, KBDC fully mediate the association between AI use and student creativity in our model. Specifically, AI use is indirectly associated with student creativity through students’ knowledge acquisition, knowledge absorptive, and knowledge combination capabilities. Third, AI self-efficacy strengthens the positive relationship between AI use and all three dimensions of KBDC. By contrast, AI trust shows a more differentiated moderating pattern. AI trust weakens the positive effects of AI use on knowledge acquisition and absorptive capabilities, but its moderating effect on knowledge combination capability is not significant.

This differentiated pattern suggests that the constraining role of AI trust operates differently across the three dimensions of KBDC. More specifically, knowledge acquisition capability involves identifying and obtaining useful external knowledge, whereas knowledge absorptive capability involves assimilating and exploiting that knowledge (Zheng et al., 2011). These two capabilities depend more directly on active searching, selection, and understanding of external information. When students place excessive trust in AI, they become more prone to uncritical reliance and thoughtless use of AI, which can reduce their motivation to search for, verify, and deeply process external knowledge (Hou et al., 2025; Romeo and Conti, 2026). Under such conditions, the positive association between AI use and these two capabilities may therefore become weaker.

By contrast, knowledge combination capability captures the ability to bring together, integrate, and apply internal and external knowledge (Zheng et al., 2011). It reflects a more active process in which students recombine AI-provided information with what they already know and rework it into new value. In this sense, knowledge combination is not a passive reception process but a higher-order, reconfigurative capability that depends more on students’ active cognitive engagement in reorganizing and applying knowledge (Denford, 2013; Kaur, 2023). Thus, the positive association between AI use and knowledge combination capability appears less vulnerable to the constraining role of AI trust. An alternative explanation for this differentiated moderating pattern may lie in how AI trust was measured, which we discuss further in the limitations section.

Taken together, this finding suggests that the constraining role of AI trust may not operate uniformly across all dimensions of KBDC. Instead, it may be more relevant to earlier-stage knowledge processes, such as knowledge acquisition and absorption, than to higher-order knowledge combination. In this sense, the result qualifies our proposed theoretical model by indicating that the boundary effect of AI trust is dimension-specific across KBDC rather than universal.

### 5.2 Theoretical contributions

Despite the rapid adoption of artificial intelligence (AI) tools in education, we still know relatively little about how AI use translates into student creative outcomes and under what conditions these effects emerge (Habib et al., 2024; Mazhar and Arseven, 2026). This study responds to this question and makes three main theoretical contributions.

First, our primary theoretical contribution lies in identifying a more fine-grained, capability-based mediating mechanism underlying the relationship between AI use and student creativity. Existing research on AI and creativity in educational settings has primarily examined how students use AI (Chan and Hu, 2023), how AI can support learning, such as through ideation support (Habib et al., 2024), and whether its effects on creative outcomes are ultimately beneficial or harmful (Rasul et al., 2023). This literature has gradually shifted away from treating AI as having a simple direct effect on creativity, and has begun to explain this relationship in more process-oriented terms. However, these process explanations remain relatively broad, for example, by focusing on how AI provides learning support (Chan and Hu, 2023), shapes stages of the creative process (Habib et al., 2024), and facilitates iterative human–AI interaction in ways that may promote creativity (Dai et al., 2023; Liang et al., 2023). Our study extends this literature by moving beyond these broad process accounts to develop a more specific, capability-based knowledge-processing account. More specifically, our findings suggest that the relationship between AI use and student creativity can be understood in terms of three KBDC dimensions: knowledge acquisition capability, knowledge absorptive capability, and knowledge combination capability. In doing so, our study suggests that AI may be associated with creativity not by directly producing it, but by supporting students’ ability to acquire, absorb, and recombine knowledge in ways that facilitate creative idea development.

Second, this study advances research on AI use by offering a more integrated and differentiated account of when AI use is more or less beneficial. Existing research has already recognized that AI effects are conditional on AI-related beliefs and contextual factors rather than uniform (Zhang et al., 2025). Prior studies have begun to examine AI self-efficacy and AI trust as important moderators in AI-related processes and outcomes, such as AI self-efficacy in AI adoption processes and work-related outcomes (Chang et al., 2024; Leong et al., 2025) and AI trust in learning-related outcomes (Chiu, 2025). However, these boundary conditions have largely been examined in isolation and in relation to different outcomes. Our study advances this literature by integrating AI self-efficacy and AI trust into the same capability-based process model and by indicating that they appear to operate differently: AI self-efficacy more consistently strengthens the relationship between AI use and KBDC, whereas AI trust operates in a more selective, dimension-specific manner. In this way, our study moves beyond separate contingency accounts toward a more integrated explanation of when AI-supported knowledge resources are more or less likely to be associated with KBDC and, in turn, student creativity.

Third, this study contributes to the KBDC literature by extending its theoretical scope from the organizational to the individual level in an AI-enabled learning context. Existing KBDC research has predominantly examined KBDC at the firm level, focusing on how organizations acquire, generate and combine knowledge to support innovation and competitive advantage (Kaur, 2023; Mikalef et al., 2019). Meanwhile, prior work has also implied that KBDC is not a purely macro-level phenomenon, because it emerges from the micro-foundations of organizational-level dynamic capabilities (Kaur, 2023). However, this micro-foundational perspective has rarely been extended into an explicit student-level conceptualization of KBDC. Our study advances this literature by conceptualizing students’ knowledge acquisition, absorptive, and combination capabilities as individual-level manifestations of KBDC and by situating them within AI-supported learning. In doing so, we extend KBDC beyond an organizational innovation framework to an individual-level capability framework for explaining how AI-supported knowledge work becomes associated with student creativity.

### 5.3 Practical implications

The increasing integration of artificial intelligence (AI) into higher education raises important questions about how these technologies can be used to foster meaningful learning and creative development. Building on the insights of this study, several practical implications can be drawn for educators, higher education institutions, students, and policymakers seeking to harness AI in educational contexts.

First, given that KBDC fully mediated the association between AI use and student creativity in our model, educators should not simply encourage students to use AI more frequently. Instead, they could guide students to use AI in ways that strengthen their knowledge acquisition, absorptive, and combination capabilities. For example, educators can integrate AI tools into learning tasks that require students to go through different stages of knowledge processing. Rather than simply asking students to obtain answers from AI, such tasks can ask them to identify relevant information, determine which inputs are useful, and use those inputs to construct their own arguments or creative solutions. These activities are also consistent with the findings of previous research, which indicates that AI can support learning and creativity when students actively engage with and evaluate the information it generates, rather than passively accepting it (Habib et al., 2024; Liang et al., 2023).

Second, based on our finding that AI self-efficacy strengthens the positive relationship between AI use and all three dimensions of KBDC, we believe that students need both access to AI tools and the confidence and skills to use them effectively. Prior research has similarly shown that AI self-efficacy plays an important role in shaping individuals’ engagement with AI technologies and work-related outcomes (Chang et al., 2024; Leong et al., 2025). Universities could therefore provide structured training that helps students develop AI-use competence. Such training may include how to write effective prompts, ask follow-up questions, and use AI tools to clarify or expand understanding of a problem. This can be particularly useful for students with lower AI self-efficacy, who may find it more difficult to translate AI use into effective knowledge acquisition, absorption, and combination.

Third, given the differentiated moderating effect of AI trust, we recommend that educators foster critical trust in AI among students. In our study, AI trust weakened the positive associations between AI use and knowledge acquisition and absorptive capabilities, indicating that excessive trust may reduce students’ motivation to actively search for and deeply process external knowledge. This aligns with prior research showing that inappropriate trust in AI may encourage over-reliance and reduce individuals’ own monitoring and verification efforts (Hou et al., 2025; Romeo and Conti, 2026). Therefore, instructors should encourage students to question AI-generated information and check its accuracy against reliable sources. Our findings also encourage students to view AI outputs as starting points for further thinking, rather than as final answers. Students can benefit more from AI when they actively question their accuracy before relying on them.

Meanwhile, since AI trust did not significantly moderate the relationship between AI use and knowledge combination capability, educators are advised to design learning activities that help students actively recombine and apply knowledge. For instance, assignments could ask students to explain how they have revised AI-generated information, connected it with their prior knowledge, and used it to develop original ideas. For policymakers, the findings suggest that AI-related educational policies need to go beyond expanding access to AI tools. Policy efforts should create the conditions for effective and responsible AI-supported learning, such as through AI literacy training and guidance on appropriate AI use. Overall, these implications suggest that the educational value of AI depends less on the use of AI itself and more on how it is integrated into learning practices. AI is more likely to enhance creativity when students are guided to use it critically and actively as part of a knowledge and capability building process rather than as a substitute for independent thinking.

### 5.4 Limitations and future directions

This study has four limitations that also suggest fruitful directions for future research. First, our data were drawn from university students in China, and the findings may therefore reflect the cultural and institutional specificity of the Chinese higher education context. Prior cross-national research suggests that students’ AI use and attitudes may vary across higher education systems due to cultural and institutional differences (Strzelecki and ElArabawy, 2024; Vaněček et al., 2025). Thus, the extent to which students use AI and the way such use relates to knowledge processing and creativity may be shaped by context-specific factors, such as assessment norms. Future research could apply our model across different national and institutional settings to examine whether these relationships vary under distinct educational norms and AI-use environments.

Second, although this study responds to the mixed findings in prior research by explaining one important pathway through which AI use is associated with student creativity, it does not fully reconcile all conflicting findings in the literature. Future research could examine whether the relationship between AI use and student creativity is nonlinear, which may provide further insight into why prior studies have reached divergent conclusions. In addition, while this study provides a focused explanatory framework, it does not capture the full range of conditions under which AI use may foster or hinder creativity. Future work could broaden the model by considering additional moderators, such as AI literacy and task complexity, to develop a more refined understanding of when AI use is most conducive to creativity.

Third, the cross-sectional design of this study limits strong causal inference. Although we conducted an additional reverse causality check and found that student creativity and the three KBDC dimensions did not significantly predict AI use, this analysis cannot fully rule out alternative causal explanations. In addition, some unobserved factors, such as learning motivation, AI literacy, and academic ability, may influence both AI use and creativity. Although we included additional learning-related control variables, namely exploratory learning and exploitative learning, our data did not allow us to control for all potentially relevant factors. Future research could therefore adopt longitudinal, experimental, or cross-lagged panel designs and include a broader set of individual learning-related variables to provide stronger evidence regarding causal direction and to better address potential endogeneity concerns.

Another limitation relates to the measurement of AI trust. While our measure captured students’ general trust in AI, it may not have fully reflected stronger forms of maladaptive reliance, such as overreliance or uncritical dependence. This may partly explain why the moderating effect of AI trust on knowledge combination capability was not significant. Future research could therefore distinguish more clearly between trust in AI and maladaptive reliance on AI, and examine whether these constructs exert different moderating effects across the dimensions of KBDC.

## Data Availability

The raw data supporting the conclusions of this article will be made available by the authors, without undue reservation.

## References

[ref1] Abduljawad S. A. (2024). Investigating the impact of ChatGPT as an AI tool on ESL writing: prospects and challenges in Saudi Arabian higher education. Int. J. Comput. Assist. Lang. Learn. Teach. 14, 1–19. doi: 10.4018/IJCALLT.367276

[ref2] Almheiri H. M. Ahmad S. Z. Khalid K. Ngah A. H. (2025). Examining the impact of artificial intelligence capability on dynamic capabilities, organizational creativity and organization performance in public organizations. J. Syst. Inf. Technol. 27, 1–20. doi: 10.1108/jsit-10-2022-0239

[ref3] Arts S. Fleming L. (2018). Paradise of novelty—or loss of human capital? Exploring new fields and inventive output. Organ. Sci. 29, 1074–1092. doi: 10.1287/orsc.2018.1216, 19642375

[ref4] Baer M. (2010). The strength-of-weak-ties perspective on creativity: a comprehensive examination and extension. J. Appl. Psychol. 95, 592–601. doi: 10.1037/a0018761, 20476837

[ref5] Baltà-Salvador R. el-Madafri I. Brasó-Vives E. Peña M. (2025). Empowering engineering students through artificial intelligence (AI): blended human–AI creative ideation processes with ChatGPT. Comput. Appl. Eng. Educ. 33, 1–19. doi: 10.1002/cae.22817, 41531421

[ref6] Bandura A. (1982). Self-efficacy mechanism in human agency. Am. Psychol. 37, 122–147. doi: 10.1037/0003-066x.37.2.122

[ref7] Bandura A. (1999). Social cognitive theory: an agentic perspective. Asian J. Social Psych. 2, 21–41. doi: 10.1111/1467-839x.00024 11148297

[ref8] Bensalah H. Mâţă L. (2022). The transition from knowledge acquisition to competency development: creativity as 21st century skill within the educational framework. J. Innov. Psychol.Educ. Didact. 26, 241–250.

[ref9] Bewersdorff A. . (2025). AI advocates and cautious critics: how AI attitudes, AI interest, use of AI, and AI literacy build university students' AI self-efficacy. Comput. Educ. Artif. Intell. 8:100340. doi: 10.1016/j.caeai.2024.100340

[ref10] Brown S. P. Ganesan S. Challagalla G. (2001). Self-efficacy as a moderator of information-seeking effectiveness. J. Appl. Psychol. 86, 1043–1051. doi: 10.1037/0021-9010.86.5.1043, 11596798

[ref11] Chan C. K. Y. Hu W. (2023). Students’ voices on generative AI: perceptions, benefits, and challenges in higher education. Int. J. Educ. Technol. High. Educ. 20:43. doi: 10.1186/s41239-023-00411-8

[ref12] Chang P. C. Zhang W. Cai Q. Guo H. (2024). Does AI-driven technostress promote or hinder employees’ artificial intelligence adoption intention? A moderated mediation model of affective reactions and technical self-efficacy. Psychol. Res. Behav. Manag. 17, 413–427. doi: 10.2147/PRBM.S441444, 38343429 PMC10859089

[ref13] Chen L. Xu M. Liu S. (2025). The dual impact pathways of generative AI use in the workplace on employee creativity. Chin. J. Manag. 22, 326–335.

[ref14] Chiu M. L. (2025). Exploring user awareness and perceived usefulness of generative AI in higher education: the moderating role of trust. Educ. Inf. Technol. 30, 21317–21351. doi: 10.1007/s10639-025-13612-7

[ref15] Chowdhury S. Budhwar P. Dey P. K. Joel-Edgar S. Abadie A. (2022). AI-employee collaboration and business performance: integrating knowledge-based view, socio-technical systems and organisational socialisation framework. J. Bus. Res. 144, 31–49. doi: 10.1016/j.jbusres.2022.01.069

[ref16] Chun Z. Ning Y. Chen J. Wijaya T. T. (2025). Exploring the interplay among artificial intelligence literacy, creativity, self-efficacy, and academic achievement in college students: findings from PLS-SEM and FsQCA. Educ. Inf. Technol. 30, 21283–21316. doi: 10.1007/s10639-025-13617-2

[ref17] Cohen W. M. Levinthal D. A. (1990). Absorptive capacity: a new perspective on learning and innovation. Adm. Sci. Q. 35, 128–152. doi: 10.2307/2393553

[ref18] Cui Y. L. Zeng M. L. Du X. K. He W. M. (2025). What shapes learners’ trust in AI? A meta-analytic review of its antecedents and consequences. IEEE Access 13, 164008–164025. doi: 10.1109/access.2025.3611367

[ref19] Dai Y. Liu A. Lim C. P. (2023). Econceptualizing ChatGPT and generative AI as a student-driven innovation in higher education. Procedia CIRP. 119, 84–90. doi: 10.1016/j.procir.2023.05.002

[ref20] Denford J. S. (2013). Building knowledge: developing a knowledge-based dynamic capabilities typology. J. Knowl. Manag. 17, 175–194. doi: 10.1108/13673271311315150

[ref21] Fan M. Cai W. (2022). How does a creative learning environment foster student creativity? An examination on multiple explanatory mechanisms. Curr. Psychol. 41, 4667–4676. doi: 10.1007/s12144-020-00974-z

[ref22] Farmer S. M. Tierney P. Kung-McIntyre K. (2003). Employee creativity in Taiwan: an application of role identity theory. Acad. Manag. J. 46, 618–630. doi: 10.2307/30040653

[ref23] Ge B. Tan L. Sheng F. Ma H. (2016). An empirical study on the relationship among innovation culture, ambidextrous learning, and dynamic capabilities. Stud. Sci. Sci. 34, 630–640.

[ref24] Glikson E. Woolley A. W. (2020). Human trust in artificial intelligence: review of empirical research. Acad. Manag. Ann. 14, 627–660. doi: 10.5465/annals.2018.0057

[ref25] Habib S. Vogel T. Anli X. Thorne E. (2024). How does generative artificial intelligence impact student creativity. J. Creat. 34:100072. doi: 10.1016/j.yjoc.2023.100072

[ref26] Haenlein M. Kaplan A. (2019). A brief history of artificial intelligence: on the past, present, and future of artificial intelligence. Calif. Manag. Rev. 61, 5–14. doi: 10.1177/0008125619864925

[ref27] Han Y. Li D. (2015). Effects of intellectual capital on innovative performance: the role of knowledge-based dynamic capability. Manag. Decis. 53, 40–56. doi: 10.1108/MD-08-2013-0411

[ref28] Han L. Zhao Y. (2025). The SECI model of knowledge creation as an enabler of students’ creative behavior through the lens of absorptive capacity. Front. Psychol. 16:1708055. doi: 10.3389/fpsyg.2025.1708055, 41583761 PMC12827523

[ref29] Hoff K. A. Bashir M. (2015). Trust in automation: integrating empirical evidence on factors that influence trust. Hum. Factors 57, 407–434. doi: 10.1177/0018720814547570, 25875432

[ref30] Hou C. Zhu G. Sudarshan V. (2025). The role of critical thinking on undergraduates' reliance behaviours on generative AI in problem-solving. Br. J. Educ. Technol. 56, 1919–1941. doi: 10.1111/bjet.13613

[ref9001] Henseler J. Ringle C. M. Sarstedt M. (2015). A new criterion for assessing discriminant validity in variance-based structural equation modeling. J. Acad. Mark. Sci. 43, 115–135. doi: 10.1007/s11747-014-0403-8

[ref31] Hsu M. H. Ju T. L. Yen C.-H. Chang C.-M. (2007). Knowledge sharing behavior in virtual communities: the relationship between trust, self-efficacy, and outcome expectations. Int. J. Hum. Comput. Stud. 65, 153–169. doi: 10.1016/j.ijhcs.2006.09.003

[ref32] Hu L. Wang H. Xin Y. (2025). Factors influencing Chinese pre-service teachers' adoption of generative AI in teaching: an empirical study based on UTAUT2 and PLS-SEM. Educ. Inf. Technol. 30, 12609–12631. doi: 10.1007/s10639-025-13353-7

[ref33] Huang C.-E. (2020). Discovering the creative processes of students: multi-way interactions among knowledge acquisition, sharing and learning environment. J. Hosp. Leis. Sport Tour. Educ. 26:100237. doi: 10.1016/j.jhlste.2019.100237, 38826717

[ref34] Huang Y. Zhang Z. Xu B. Zhou X. Zhai J. Gao D. (2025). Digital learning empowering sustainable education: evidence from the determinants of Chinese college students’ knowledge innovation capability. Sustainability 17:9060. doi: 10.3390/su17209060

[ref35] Jarrahi M. H. (2019). In the age of the smart artificial intelligence: AI’S dual capacities for automating and informating work. Bus. Inf. Rev. 36, 178–187. doi: 10.1177/0266382119883999

[ref36] Jarrahi M. H. Askay D. Eshraghi A. Smith P. (2023). Artificial intelligence and knowledge management: a partnership between human and AI. Bus. Horiz. 66, 87–99. doi: 10.1016/j.bushor.2022.03.002

[ref37] Kaur V. (2023). Knowledge-based dynamic capabilities: a scientometric analysis of marriage between knowledge management and dynamic capabilities. J. Knowl. Manag. 27, 919–952. doi: 10.1108/jkm-02-2022-0112

[ref38] Khansa L. Kuem J. Siponen M. Kim S. S. (2017). To cyberloaf or not to cyberloaf: the impact of the announcement of formal organizational controls. J. Manag. Inf. Syst. 34, 141–176. doi: 10.1080/07421222.2017.1297173

[ref39] Kock N. (2017). Common Method Bias: A Full Collinearity Assessment Method for PLS-SEM. PLS-PM, Cham, Switzerland: Springer International Publishing. 245–257.

[ref40] Kong H. Yin Z. Baruch Y. Yuan Y. (2023). The impact of trust in AI on career sustainability: the role of employee–AI collaboration and protean career orientation. J. Vocat. Behav. 146:103928. doi: 10.1016/j.jvb.2023.103928

[ref41] Kulal A. (2025). Cognitive risks of AI: literacy, trust, and critical thinking. J. Comput. Inf. Syst. 1–13. doi: 10.1080/08874417.2025.2582050

[ref42] Lane P. J. Salk J. E. Lyles M. A. (2001). Absorptive capacity, learning, and performance in international joint ventures. Strateg. Manag. J. 22, 1139–1161. doi: 10.1002/smj.206

[ref43] Lennerts S. Schulze A. Tomczak T. (2020). The asymmetric effects of exploitation and exploration on radical and incremental innovation performance: an uneven affair. Eur. Manag. J. 27, 1366–1389. doi: 10.1016/j.emj.2019.06.002

[ref44] Leong F. T. L. Li X. Chen E. M. (2025). The relationship between career adaptability and work engagement among young Chinese workers: mediating role of job satisfaction and moderating effects of artificial intelligence self-efficacy and anxiety. Behav. Sci. 15:1682. doi: 10.3390/bs15121682, 41464025 PMC12729327

[ref45] Liang T.-P. You J.-J. Liu C.-C. (2010). A resource-based perspective on information technology and firm performance: a meta analysis. Ind. Manag. Data Syst. 110, 1138–1158. doi: 10.1108/02635571011077807

[ref46] Liang J. Wang L. Luo J. Yan Y. Fan C. (2023). The relationship between student interaction with generative artificial intelligence and learning achievement: serial mediating roles of self-efficacy and cognitive engagement. Front. Psychol. 14:1285392. doi: 10.3389/fpsyg.2023.1285392, 38187430 PMC10766754

[ref47] Lin X. Wu D. (2015). AI technology adoption, knowledge sharing, and manufacturing firms' innovation performance: the moderating effect of absorptive capacity. IEEE Trans. Eng. Manag. 72, 2137–2149. doi: 10.1109/tem.2025.3573176

[ref48] Lin C. Yu C. Wu D. (2015). Effects of exploratory and exploitative learning on enterprise's disruptive innovation. R D Manag. 27, 19–28.

[ref49] Liu C. Liao Q. Lu J. (2026a). Employees’ trust in AI and innovative behavior: a JD-R model perspective. Behav. Sci. 16:425. doi: 10.3390/bs16030425, 41898086 PMC13024477

[ref50] Liu Z. Huang Y. Pan X. Yu Z. Zhou R. (2026). Taking the initiative and fostering new opportunities: research on the relationship between artificial intelligence awareness and employee creativity. J. Ind. Eng. Eng. Manag. 40, 30–43.

[ref51] Lockey S. Gillespie N. Holm D. Someh I. A. (2021). A Review of Trust in Artificial Intelligence: Challenges, Vulnerabilities and Future Directions HICSS, Atlanta, GA: Association for Information Systems. 5463–5472.

[ref52] Man Tang P. Koopman J. McClean S. T. Zhang J. H. Li C. H. De Cremer D. . (2022). When conscientious employees meet intelligent machines: an integrative approach inspired by complementarity theory and role theory. Acad. Manag. J. 65, 1019–1054. doi: 10.5465/amj.2020.1516

[ref53] Marrone R. Taddeo V. Hill G. (2022). Creativity and artificial intelligence—a student perspective. J. Intelligence 10, 1–11. doi: 10.3390/jintelligence10030065, 36135606 PMC9504190

[ref54] Mazhar B. Arseven T. (2026). Students’ writing skills and creativity in the age of artificial intelligence: a systematic review of creativity across diverse AI-supported writing contexts. Think. Skills Creat. 60:102118. doi: 10.1016/j.tsc.2025.102118

[ref55] Mikalef P. Boura M. Lekakos G. Krogstie J. (2019). Big data analytics capabilities and innovation: the mediating role of dynamic capabilities and moderating effect of the environment. Br. J. Manage. 30, 272–298. doi: 10.1111/1467-8551.12343

[ref56] Neirotti P. Pesce D. Battaglia D. (2021). Algorithms for operational decision-making: an absorptive capacity perspective on the process of converting data into relevant knowledge. Technol. Forecast. Soc. Change 173:121088. doi: 10.1016/j.techfore.2021.121088

[ref57] Podsakoff P. M. MacKenzie S. B. Lee J.-Y. Podsakoff N. P. (2003). Common method biases in behavioral research: a critical review of the literature and recommended remedies. J. Appl. Psychol. 88, 879–903. doi: 10.1037/0021-9010.88.5.879 14516251

[ref58] Prieto I. M. Easterby-Smith M. (2006). Dynamic capabilities and the role of organizational knowledge: an exploration. Eur. J. Inf. Syst. 15, 500–510. doi: 10.1057/palgrave.ejis.3000642

[ref59] Rasul T. Nair S. R. Kalendra D. Robin M. de Oliveira Santini F. Ladeira W. J. . (2023). The role of ChatGPT in higher education: benefits, challenges, and future research directions. J. Appl. Learn. Teach. 6, 41–56. doi: 10.37074/jalt.2023.6.1.29

[ref60] Romeo G. Conti D. (2026). Exploring automation bias in human–AI collaboration: a review and implications for explainable AI. AI Soc 41, 259–278. doi: 10.1007/s00146-025-02422-7

[ref61] Ruffino C. Gueugneau N. Grosprêtre S. (2025). Barriers and facilitators to sports participation in autistic Europeans: insights from a large-scale questionnaire survey. Front. Sports Act. Living 7:1580462. doi: 10.3389/fspor.2025.1580462, 40740882 PMC12307315

[ref62] Runco M. A. Jaeger G. J. (2012). The standard definition of creativity. Creat. Res. J. 24, 92–96. doi: 10.1080/10400419.2012.650092

[ref63] Schweder S. (2019). The role of control strategies, self-efficacy, and learning behavior in self-directed learning. Int. J. School Educ. Psychol. 7, 29–41. doi: 10.1080/21683603.2018.1459991

[ref64] Shahzad M. Shahzad M. F. Xu S. Zahid H. (2025). Exploring the impact of generative AI-based technologies on learning performance through self-efficacy, fairness & ethics, creativity, and trust in higher education. Educ. Inf. Technol. 30, 3691–3716. doi: 10.1007/s10639-024-12949-9

[ref65] Shan B. Liu K. Lu X. Liu X. (2025). Artificial intelligence, knowledge coupling, and dynamic capabilities in China’s GEM listed enterprises: the role of human–AI collaboration. J. Knowl. Manag., 1–22. doi: 10.1108/JKM-04-2025-0588, 35579975

[ref66] Shao Z. Li X. Wang Q. (2022). From ambidextrous learning to digital creativity: an integrative theoretical framework. Inf. Syst. J. 32, 544–572. doi: 10.1111/isj.12361

[ref67] Singh J. Fleming L. (2010). Lone inventors as sources of breakthroughs: myth or reality. Manag. Sci. 56, 41–56. doi: 10.1287/mnsc.1090.1072, 19642375

[ref68] Stadnik M. Lokas Ćošković A. Banko T. Ružak D. (2025). AI and writing skills: students' attitudes towards using AI to enhance their writing based on the example of algebra university students. Interdiscip. Descr. Complex Syst. 23, 46–71. doi: 10.7906/indecs.23.1.3

[ref69] Strzelecki A. ElArabawy S. (2024). Investigation of the moderation effect of gender and study level on the acceptance and use of generative AI by higher education students: comparative evidence from Poland and Egypt. Br. J. Educ. Technol. 55, 1209–1230. doi: 10.1111/bjet.13425

[ref70] Sundaresan S. Zhang Z. (2021). AI-enabled knowledge sharing and learning: redesigning roles and processes. Int. J. Organ. Anal. 30, 983–999. doi: 10.1108/ijoa-12-2020-2558, 35579975

[ref71] Teodoridis F. Bikard M. Vakili K. (2019). Creativity at the knowledge frontier: the impact of specialization in fast- and slow-paced domains. Adm. Sci. Q. 64, 894–927. doi: 10.1177/0001839218793384

[ref72] Toth Z. Goga A.-S. Boșcoianu M. (2025). AI integration in fundamental logistics components: advanced theoretical framework for knowledge process capabilities and dynamic capabilities hybridization. Logistics 9:140. doi: 10.3390/logistics9040140

[ref73] Vaněček D. Yorulmaz Y. I. Dobrovská D. (2025). A holistic exploration of student attitudes toward AI use in higher education: an international comparison. Cogent Educ. 12:2571691. doi: 10.1080/2331186X.2025.2571691, 37339054

[ref74] Vătămănescu E. M. Bratianu C. Dabija D.-C. Popa S. (2023). Capitalizing online knowledge networks: from individual knowledge acquisition towards organizational achievements. J. Knowl. Manag. 27, 1366–1389. doi: 10.1108/jkm-04-2022-0273

[ref75] Wang J. Li Y. (2023). Cultivating top innovative talent in the wave of ChatGPT: value implications, practical concerns, and ecological reconstruction. China Educ. Technol. 11, 62–71.

[ref76] Wang M. Yang Y. Zhong P. (2025). How does AI affect the self-actualization of content creators in dynamic environments? A knowledge management perspective. Technol. Soc. 81:102855. doi: 10.1016/j.techsoc.2025.102855, 38826717

[ref77] Wang R. Zhang Z. Zhao W. Li S. Pan Y. (2026). Exploring the relationship between generative AI usage and employee creativity: a dual-pathway mediation model. Eur. J. Innov. Manag. 29, 799–823. doi: 10.1108/ejim-02-2024-0213

[ref78] Wei W. Liu B. N. Huang W. (2022). The double-edged effect of job gamification on job involvement of gig workers: the role of flow experience and overwork. Nankai Bus. Rev. 25, 159–171.

[ref79] Yam K. C. Tang P. M. Jackson J. C. Su R. Gray K. (2023). The rise of robots increases job insecurity and maladaptive workplace behaviors: multimethod evidence. J. Appl. Psychol. 108, 850–870. doi: 10.1037/apl0001045, 36222634

[ref80] Yi J. B. Zhang Z. Y. Yang X. P. Wang Y. T. (2022). Internet enterprise organizational inertia, digital capability and business model innovation. Nankai Bus. Rev. 25, 29–42.

[ref81] Yu H. (2023). Reflection on whether chat GPT should be banned by academia from the perspective of education and teaching. Front. Psychol. 14:1181712. doi: 10.3389/fpsyg.2023.1181712, 37325766 PMC10267436

[ref82] Zahedi F. M. Song J. (2014). Dynamics of trust revision: using health infomediaries. J. Manag. Inf. Syst. 24, 225–248. doi: 10.2753/mis0742-1222240409

[ref83] Zahra S. A. George G. (2002). Absorptive capacity: a review, reconceptualization, and extension. Acad. Manag. Rev. 27, 185–203. doi: 10.2307/4134351

[ref84] Zhang L.-X. Li J.-M. Wang L.-L. Mao M.-Y. Zhang R.-X. (2023). How does the usage of robots in hotels affect employees’ turnover intention? A double-edged sword study. J. Hosp. Tour. Manag. 57, 74–83. doi: 10.1016/j.jhtm.2023.09.004

[ref85] Zhang Q. Liao G. Ran X. Wang F. (2025). The impact of ai usage on innovation behavior at work: the moderating role of openness and job complexity. Behav. Sci. 15:491. doi: 10.3390/bs15040491, 40282112 PMC12024388

[ref86] Zhang Z. G. Fu S. Y. Yu C. P. (2018). The effect of individual knowledge absorptive capacity on employee innovation performance. Hum. Resour. Dev. China 35, 73–83. doi: 10.16471/j.cnki.11-2822/c.2018.03.007

[ref87] Zheng S. Zhang W. Du J. (2011). Knowledge-based dynamic capabilities and innovation in networked environments. J. Knowl. Manag. 15, 1035–1051. doi: 10.1108/13673271111179352

[ref88] Zheng S. L. Zhang W. Wu X. B. (2010). Knowledge-based dynamic capabilities: theory and empirical study. Stud. Sci. Sci. 28, 405–411+466.

[ref89] Zhou M. Peng S. (2025). The usage of AI in teaching and students' creativity: the mediating role of learning engagement and the moderating role of AI literacy. Behav. Sci. 15:587. doi: 10.3390/bs15050587, 40426365 PMC12109026

